# Treating Aortic Stenosis in Young Patients

**DOI:** 10.1016/j.jacadv.2024.101311

**Published:** 2024-09-27

**Authors:** Sivakumar Sudhakaran, Vinod H. Thourani, Mayra E. Guerrero

**Affiliations:** a Department of Cardiovascular Medicine, Mayo Clinic, Rochester, Minnesota, USA; b Department of Cardiovascular Surgery, Marcus Valve Center, Piedmont Heart Institute, Atlanta, Georgia, USA

**Keywords:** aortic stenosis, lifetime management, low-risk, SAVR, TAVR, valve, -in-valve, young patients

There has been a significant increase in transcatheter aortic valve replacement (TAVR) volumes in the last decade. While initial trials compared TAVR to medical therapy in extreme risk patients and eventually to surgical aortic valve replacement (SAVR) in high and intermediate-risk patients, more contemporary randomized trials evaluated the safety and efficacy of TAVR in younger low-risk patients.^1^^,^^2^ In 2019, randomized trials found that TAVR with self-expandable transcatheter heart valve (THV) was noninferior to SAVR for the primary composite endpoint of death or disabling stroke at 24 months,^2^ and TAVR with a balloon-expandable THV was superior to SAVR for the primary composite endpoint of death, stroke and valve-related hospitalization at 1 year.^1^ The mean age and Society of Thoracic Surgeons predicted risk of mortality score of the patients enrolled in the TAVR arm of the balloon-expandable and self-expandable THV low-risk trials were 73.3% ± 5.8 years and 1.9% ± 0.7%^1^ and 74.1 ± 5.8 years and 1.9% ± 0.7%,^2^ respectively. Based on these trials, the American College of Cardiology/American Heart Association consensus guidelines have recommended shared decision making regarding TAVR versus SAVR in patients between 65 to 80 years old with severe symptomatic aortic stenosis. SAVR was given a Class I indication for patients <65 years of age with life expectancy greater than 20 years.^3^

Despite this, recent data has shown a steady increase in TAVR utilization volumes in patients <65 years of age, with a corresponding decrease in SAVR.^4^ Recent retrospective data presented at the 2024 Society of Thoracic Surgeons scientific meeting compared outcomes for patients <60 years of age undergoing surgery and those undergoing TAVR in California between 2013 and 2021.^5^ By 2021, almost half of patients younger than 60 years were treated with TAVR instead of SAVR. Moreover, while 30-day mortality rates were similar, 5-year survival rates were higher in patients treated with SAVR compared to TAVR.^5^ These findings are from nonrandomized retrospective data with multiple potential confounders that could remain present despite propensity matching analysis. Therefore, a prospective randomized study is needed to better understand the safety and efficacy of TAVR in this low-risk younger patient population. This study would address additional important knowledge gaps, including longer term mortality and valve performance durability data, in addition to the management of subsequent aortic valve interventions when bioprosthetic valve failure occurs.

Longer term data are emerging from the PARTNER 3 (Placement of Aortic Transcatheter Valve 3) and Evolut Low Risk clinical trials following outcomes in low-surgical risk patients who received TAVR versus SAVR. Both major commercially available transcatheter systems thus far have been noninferior to SAVR in terms of mortality,^6^^,^^7^ and at 4 years the self-expanding TAVR system has shown a statistically significant relative reduction in hazard for all-cause mortality or disabling stroke.^7^ It should be noted however, that all-cause SAVR mortality was higher in the self-expanding trial at 4 years (12.1%),^7^ than in the balloon-expandable trial at 5 years (8.2%).^6^ Rates of bioprosthetic valve degeneration (BVD) were similar between SAVR and balloon-expandable TAVR system at 5 years in low-risk patients.^6^ Long-term BVD data in low-risk patients who underwent self-expanding TAVR is pending. However, in intermediate and high-risk surgical patients, significantly less BVD was seen with self-expanding TAVR compared to SAVR at 5 years.^8^ The Nordic Aortic Valve Intervention trial randomized 280 low-risk patients with severe aortic stenosis to surgery versus 1st generation self-expanding TAVR valve and found no difference in mortality or bioprosthetic valve failure at 10 years. Notably, at 10 years, more than 40% of patients of TAVR patients in Nordic Aortic Valve Intervention required permanent pacemaker implantation compared to 14% in SAVR patients. Mild or more paravalvular leak (PVL) was also more frequent in TAVR (18%) compared to SAVR (5.2%).^9^ Though these may be findings isolated to the first-generation self-expanding valve, when considering TAVR implantation in younger patients, higher rates of implantation and PVL is concerning. More longitudinal data are needed to determine the safety and feasibility of using TAVR in patients less than 65 years old.

Although mechanical valve prostheses are more durable and recommended for young patients, the risks associated with the required long-term anticoagulation (often needing lifestyle modifications to lower such risk) are important reasons for which patients choose to be treated with a bioprosthetic valve even at a young age. In addition, the availability of transcatheter aortic valve in valve (AViV) with a TAVR procedure is another reason why many patients choose tissue valves, knowing that the repeat aortic valve replacement can be done percutaneously if/when subsequent replacement is needed. Given growing patient preference for TAVR instead of SAVR with a bioprosthesis, future randomized clinical trials are needed comparing TAVR versus bioprosthetic SAVR. Perhaps the next biggest question is regarding the durability and safety of the subsequent aortic valve replacement procedure after index procedure. At least 20-year follow-up would be needed to better characterize this.

How do the safety profiles of TAVR in SAVR or TAVR in TAVR compare, and how durable is the result? Moreover, in young patients who receive TAVR as an index procedure, are they better served with repeat TAVR in TAVR or perhaps with surgical excision of existing TAVR and replacement with surgical bioprosthetic? Studying these outcomes will become even more complex as we move on to 4th generation balloon-expandable and self-expanding TAVR systems, in addition to newer TAVR devices coming to market. Permanent pacemakers and PVL will be of greater clinical consequence in younger patients and longer-term outcomes must be followed. Difficulties with coronary access has been a known problem with transcatheter valves, especially with the taller self-expanding THV currently available, and over time may be a significant cause of morbidity and mortality. Registry based retrospective cohort data found no difference in mortality or stroke in low surgical risk patients who underwent TAVR for bicuspid aortic stenosis compared with tricuspid aortic stenosis.^10^ However, TAVR in bicuspid aortic valves has not been evaluated in randomized clinical trials. Young patients with suitable bicuspid valves for either TAVR or SAVR should be included to study the safety and efficacy of TAVR in these patients.

In summary, despite consensus guidelines, TAVR is already being utilized in patients <65 years of age. Nationally, we are experiencing a steady annual increase in TAVR volumes in patients <65 years of age.^4^ Longer term, prospective randomized data are needed to understand the safety and efficacy of TAVR compared to SAVR in these patients. Additionally, trial follow-up should ideally be extended to understand the best type of subsequent aortic replacement after bioprosthetic valve failure. Understanding the longevity and durability of each of these permutations such as TAVR versus SAVR, TAVR in SAVR, TAVR in TAVR, or surgical excision of TAVR with bioprosthetic replacement would become a powerful tool in counseling patients to better inform strategy for initial treatment of aortic stenosis in young patients.

## Funding support and author disclosures

The authors have reported that they have no relationships relevant to the contents of this paper to disclose.

## References

[1] Mack M.J., Leon M.B., Thourani V.H. (2019). Transcatheter aortic-valve replacement with a balloon-expandable valve in low-risk patients. N Engl J Med.

[2] Popma J.J., Deeb G.M., Yakubov S.J. (2019). Transcatheter aortic-valve replacement with a self-expanding valve in low-risk patients. N Engl J Med.

[3] Otto C.M., Nishimura R.A., Bonow R.O. (2021). 2020 ACC/AHA guideline for the management of patients with valvular heart disease: a report of the American College of Cardiology/American heart association joint committee on clinical practice guidelines. J Am Coll Cardiol.

[4] Sharma T., Krishnan A.M., Lahoud R., Polomsky M., Dauerman H.L. (2022). National trends in TAVR and SAVR for patients with severe isolated aortic stenosis. J Am Coll Cardiol.

[5] Alabbadi S., Malas J., Chen Q. (January 27-29, 2024). Guidelines versus practice: a statewide survival analysis of SAVR versus TAVR in patients aged <60 years. Presented at the 2024 Society of Thoracic Surgeons Annual Meeting.

[6] Mack M.J., Leon M.B., Thourani V.H. (2023). Transcatheter aortic-valve replacement in low-risk patients at five years. N Engl J Med.

[7] Forrest J.K., Deeb G.M., Yakubov S.J. (2023). 4-Year outcomes of patients with aortic stenosis in the Evolut low risk trial. J Am Coll Cardiol.

[8] O'Hair D., Yakubov S.J., Grubb K.J. (2023). Structural valve deterioration after self-expanding transcatheter or surgical aortic valve implantation in patients at intermediate or high risk. JAMA Cardiol.

[9] Thyregod H.G.H., Jørgensen T.H., Ihlemann N. (2024). Transcatheter or surgical aortic valve implantation: 10-year outcomes of the NOTION trial. Eur Heart J.

[10] Makkar R.R., Yoon S.H., Chakravarty T. (2021). Association between transcatheter aortic valve replacement for bicuspid vs tricuspid aortic stenosis and mortality or stroke among patients at low surgical risk. JAMA.

